# Liposomal paclitaxel versus docetaxel in induction chemotherapy using Taxanes, cisplatin and 5-fluorouracil for locally advanced nasopharyngeal carcinoma

**DOI:** 10.1186/s12885-018-5192-x

**Published:** 2018-12-20

**Authors:** Sai-Lan Liu, Xue-Song Sun, Xiao-Yun Li, Qiu-Yan Chen, Huan-Xin Lin, Yue-Feng Wen, Shan-Shan Guo, Li-Ting Liu, Hao-Jun Xie, Qing-Nan Tang, Yu-Jing Liang, Jin-Jie Yan, Chao Lin, Zhen-Chong Yang, Lin-Quan Tang, Ling Guo, Hai-Qiang Mai

**Affiliations:** 1 State Key Laboratory of Oncology in South China,Collaborative Innovation Center for Cancer Medicine, Sun Yat-sen University Cancer Center, Guangzhou, Guangdong Province People’s Republic of China; 2 Department of Nasopharyngeal Carcinoma, Sun Yat-sen University Cancer Center, Guangzhou, Guangdong Province People’s Republic of China; 3 Department of Radiotherapy, Sun Yat-sen University Cancer Center|, Guangzhou, Guangdong Province People’s Republic of China

**Keywords:** Nasopharyngeal carcinoma, Induction chemotherapy, Docetaxel, Liposomal paclitaxel

## Abstract

**Background:**

We wished to evaluate the efficacy and safety of liposomal paclitaxel and docetaxel for induction chemotherapy (IC) for nasopharyngeal carcinoma (NPC).

**Methods:**

A total of 1498 patients with newly-diagnosed NPC between 2009 and 2017 treated with IC plus concurrent chemotherapy were included in our observational study. Overall survival (OS), progression-free survival (PFS), locoregional relapse-free survival (LRFS), distant metastasis-free survival (DMFS) and grade-3–4 toxicities were compared between groups using propensity score matching (PSM).

**Results:**

In total, 767 patients were eligible for this study, with 104 (13.6%) and 663 (86.4%) receiving a liposomal paclitaxel-based and docetaxel-based taxanes, cisplatin and 5-fluorouracil (TPF) regimen, respectively. PSM identified 103 patients in the liposomal-paclitaxel group and 287 patients in the docetaxel group. There was no significant difference at 3 years for OS (92.2% vs. 93.9%, *P* = 0.942), PFS (82.6% vs. 81.7%, *P* = 0.394), LRFS (94.7% vs. 93.3%, *P* = 0.981) or DMFS (84.6% vs. 87.4%, *P* = 0.371) between the two groups after PSM. Significant interactions were not observed between the effect of chemotherapy regimen and sex, age, T stage, N stage, overall stage, or Epstein–Barr virus DNA level in the subgroup multivariate analysis. The prevalence of grade-3–4 leukopenia and neutropenia in the liposomal-paclitaxel group was significantly lower than that of the docetaxel group (*P* < 0.05 for all).

**Conclusions:**

Compared with docetaxel, liposomal paclitaxel has identical anti-tumor efficacy, but causes fewer and milder adverse reactions in IC for NPC.

## Background

Nasopharyngeal carcinoma (NPC) is a malignant disease arising from the nasopharyngeal epithelium. The incidence of NPC is particularly high in Southern China, where 50–80 cases per 100,000 persons are reported each year [1]. Because of the radiosensitive nature of NPC and the typically deep-seated location of the lesions, radiotherapy (RT) is the primary treatment for NPC [2]. Advances in radiation delivery have resulted in improved local control for NPC [3, 4]. However, prevention of distant metastasis in advanced NPC remains unsatisfactory and is the main cause of treatment failure [5, 6]. Several studies have shown that induction chemotherapy (IC) followed by definitive concurrent chemoradiotherapy (CCRT) may be associated with reduced risk of distant metastases, which could improve clinical outcomes [7–9].

Studies have shown that taxanes, cisplatin and 5-fluorouracil (TPF) is the effective IC regimen for reducing the risk of treatment failure and improving overall survival (OS) in patients with high risk NPC [10–12]. In comparison with the standard cisplatin and fluorouracil (PF) regimen, regimens involving taxanes (microtubule-stabilizing drugs) have been used widely for the treatment of different types of malignancies for decades [13].

Docetaxel is a semi-synthetic taxane-based agent that has an enhanced ability to assemble tubulin in vitro [14]. Use of docetaxel has gradually superseded that of paclitaxel and occupied the dominant position in the last decade [15]. However, due to the undesirable water solubility of docetaxel, it is formulated with Tween 80 and ethanol. As a consequence, patients must be pre-medicated by corticosteroids for days to minimize the severe hypersensitivity reactions and fluid retention brought about by solvent-based docetaxel. “Liposomal paclitaxel” is a new paclitaxel drug encapsulated with liposomes which can reduce toxicities and improve bioavailability [16, 17]. Liposomal paclitaxel has comparable antitumor efficacy with that of conventional paclitaxel [18].

As an important agent in IC regimens, the toxicities of different taxane-based analogs affect the tolerance and compliance of patients considerably, thereby influencing IC efficacy. Under these circumstances, comparing the efficacy and toxicity of liposomal paclitaxel with that of docetaxel is important. We undertook a retrospective study to evaluate efficacy and toxicity of liposomal paclitaxel and docetaxel for the treatment of locally advanced NPC.

## Methods

### Patients

The study protocol was approved by the Research Ethics Committee of the Cancer Center of Sun Yat-sen University (Guangdong, China). Newly diagnosed patients treated in the Sun Yat-sen Cancer Center from 2009 to 2017 were identified. The inclusion criteria were: NPC confirmed by histology; age ≥ 18 years; in receipt of IC and CCRT; Karnofsky Performance Score > 70; availability of hematology data and results for Epstein–Barr virus (EBV) serology. Patients being treated under the IC regimen of taxanes and cisplatin (TP) or PF or adjuvant chemotherapy, patients with stage-II NPC, or those given conventional paclitaxel were excluded.

After the screening procedure, 767 individuals were chosen for the analysis. This cohort comprised 104 patients receiving liposomal paclitaxel and 663 patients receiving docetaxel.

### Data collection

Using the medical records of patients, the following information was collected: demographics; diagnosis; tumor stage; imaging; EBV DNA results; regimen; chemotherapy dose; laboratory results at baseline and after each cycle of chemotherapy. Patients were followed up every 3 months in the first 2 years, and then every 6 months thereafter. Recurrence or metastasis was confirmed by pathology results or imaging (magnetic resonance imaging, computed tomography, abdominal ultrasound, whole-body bone scintigraphy, positron emission tomography–computed tomography). Our endpoints included overall survival (OS, the interval from the first day of hospitalization to death from any cause), progression-free survival (PFS, the interval from the first day of hospitalization to disease progression or death from any cause), locoregional relapse-free survival (LRFS) and distant metastasis-free survival (DMFS), which corresponded to the interval to first recurrence and distant metastasis, respectively. Hematologic and non-hematologic toxicities were graded under the instruction of the Common Terminology Criteria for Adverse Events v4.0.

### Induction chemotherapy and concurrent chemoradiotherapy

All patients received the TPF IC regimen: docetaxel (60 mg/m^2^, day-1) or liposomal paclitaxel (135 mg/m^2^, day-1), cisplatin (20–25 mg/m^2^/day, days 1–3), and 5-fluorouracil (500–800 mg/m^2^, 120 h of continuous intravenous infusion). All regimens were administered every 3 weeks over 2–4 cycles. RT was administered to the nasopharynx and neck using intensity-modulated radiotherapy (IMRT) or two-dimensional radiotherapy. IC was followed by concurrent cisplatin-based chemotherapy (80–100 mg/m^2^ every 3 weeks or 30–40 mg/m^2^ every week) [19, 20]. Five daily fractions of a total dose of 68–70 Gy at ≈2 Gy per fraction were prescribed per week. Other details of the IMRT plan were in accordance with the principles described previously [21–23].

### Statistical analyses

The propensity score matching (PSM) method was adopted to adjust for potential confounders that may influence estimation of the treatment effect. Propensity scores were calculated by logistic regression at 1:3 ratios to balance the covariates of sex, age, T stage, N stage, and plasma levels of EBV DNA. The chi-squared test or Fisher’s exact test was used to compare the distribution of categorical factors between the docetaxel group and liposomal-paclitaxel group in the observational dataset and PSM dataset. Survival curves and survival outcomes were analyzed using the Kaplan–Meier method and log-rank tests. A multivariable Cox proportional hazards model was used for multivariable analysis, to calculate hazard ratios (HRs) and evaluate prognostic values. *P* < 0.05 (two-sided) was considered significant. SPSS v22.0 was used for all statistical analyses.

## Results

### Patient characteristics

**Fig. 1:**
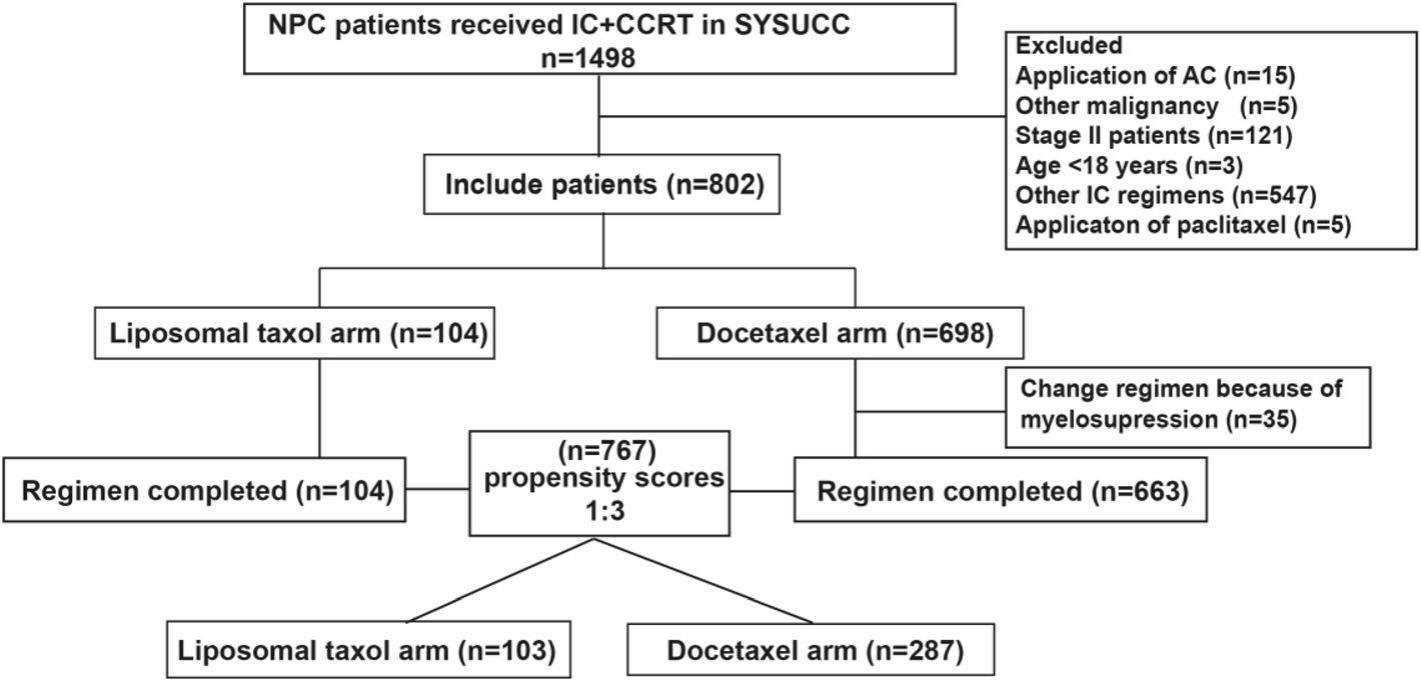
Flowchart for patient inclusion. IC, induction chemotherapy; CCRT, concurrent chemoradiotherapy; AC, adjuvant chemotherapy; PTX, paclitaxel

From 2009 to 2017, 802 patients were initially considered to be qualified for this study. There were 35 patients receiving docetaxel that were switched to other chemotherapy regimens because of severe myelosuppression after one cycle of chemotherapy so we excluded these patients. Finally, after the screening procedure, 767 patients were involved in this study; 104 (13.6%) and 663 (86.4%) patients were treated with liposomal paclitaxel and docetaxel, respectively, in IC regimens. All patients have complete full radiotherapy in both groups. There were 61(58.7%) patients receiving a cumulative cisplatin dose (CCD) ≥200 mg/m2 in the liposomal group and 119(17.9%) patients receiving a CCD ≥200 mg/m2 in the docetaxel group. After PSM at a ratio of 1:3, a well-balanced cohort of 390 patients remained in the analysis (Fig. 1). Among them, 103 patients were in the liposomal-paclitaxel group and 287 patients in the docetaxel group. The median age was 44 (18–72) years, with 91 (23.3%) females and 299 (76.7%) males. Significant differences in potential prognostic factors were not observed in these two groups (*P* > 0.05 for all). The differences in patient characteristics between the liposomal-paclitaxel and docetaxel groups in the observational and propensity-matched datasets are shown in Table 1.

**Table 1:** Difference in patient characteristics between the liposomal PTX and docetaxel groups in the observational and propensity-matched datasets

Characteristic	Observational dataset (*n* = 767)		*P*	PSM dataset (*n* = 390)		*P*
	Liposomal PTX	Docetaxel		Liposomal PTX	Docetaxel	
Total	104	663		103	287	
Age, years			0.261^a^			0.153^a^
Median (range)	43(18–74)	45(19–72)		45(19–72)	42(18–70)	
< 45	50(48.1)	358(54.0)		49(47.6)	160(55.7)	
≥ 45	54(51.9)	305(46.0)		54 (52.4)	127(44.3)	
Sex			0.795^a^			0.793^a^
Female	26(25.0)	158(23.8)		25(24.3)	66(23.0)	
Male	78(75.0)	505(76.2)		78(75.7)	221(77.0)	
Pathologic (WHO) type			1.000^b^			–
I	0(0.0)	3(0.5)		0(0)	0(0)	
II	0(0.0)	4(0.6)		0(0)	0(0)	
III	104(100)	656(98.9)		103(100)	287(100)	
T stage*			0.823^a^			0.801^a^
T1	1(1.0)	10(1.5)		1(1.0)	6(2.0)	
T2	12(11.5)	59(8.9)		12(11.7)	37(12.9)	
T3	50(48.1)	325(49.0)		50(48.5)	144(50.2)	
T4	41(39.4)	269(40.6)		40(38.8)	100(34.8)	
N stage*			0.006^a^			0.939^a^
N0	4(3.8)	10(1.5)		3(2.9)	8(2.8)	
N1	14(13.5)	179(27.0)		14(13.6)	41(14.3)	
N2	51(49.0)	313(47.2)		51(49.5)	150(52.3)	
N3	35(33.7)	161(24.3)		35(34.0)	88(30.7)	
Clinical stage		0.109^a^			0.547^a^	
III	36(34.6)	280(42.2)		36(35.0)	118(41.1)	
IVa	33(31.7)	222(33.5)		32(31.1)	81(28.2)	
IVb	35(33.7)	161(24.3)		35(34.0)	88(30.7)	
EBV DNA (copies/mL)			0.023^a^			0.825^a^
< 1500	45(43.3)	212(32.0)		44(42.7)	119(41.5)	
≥ 1500	59(56.7)	451(68.0)		59(57.3)	168(58.5)	
RT method		0.541^c^	1.000 ^c^			
2D RT	2(1.9)	5(0.8)		2(1.9)	4(1.4)	
IMRT	102(98.1)	658(99.2)		101(98.1)	283(98.6)	
Cycles of IC			0.535^a^			0.849^a^
2	29(27.9)	166(25.0)		29(28.2)	78(27.2)	
3–4	75(72.1)	497(75.0)		74(71.8)	209(72.8)	

*PTX* paclitaxel, *EBV* Epstein–Barr virus, *RT* radiotherapy, 2*DRT* two-dimensional radiotherapy, *IMRT* intensity-modulated radiotherapy, *WHO* World Health Organization, *IC* induction chemotherapy

^*a*^*P* value was calculated using the chi-square test. ^b^*P* value was calculated using Fisher’s exact test. ^c^P value was calculated by correction for continuity chi-square test

^*^According to the 7th edition of UICC/AJCC staging system

### Survival outcomes

**Fig. 2:**
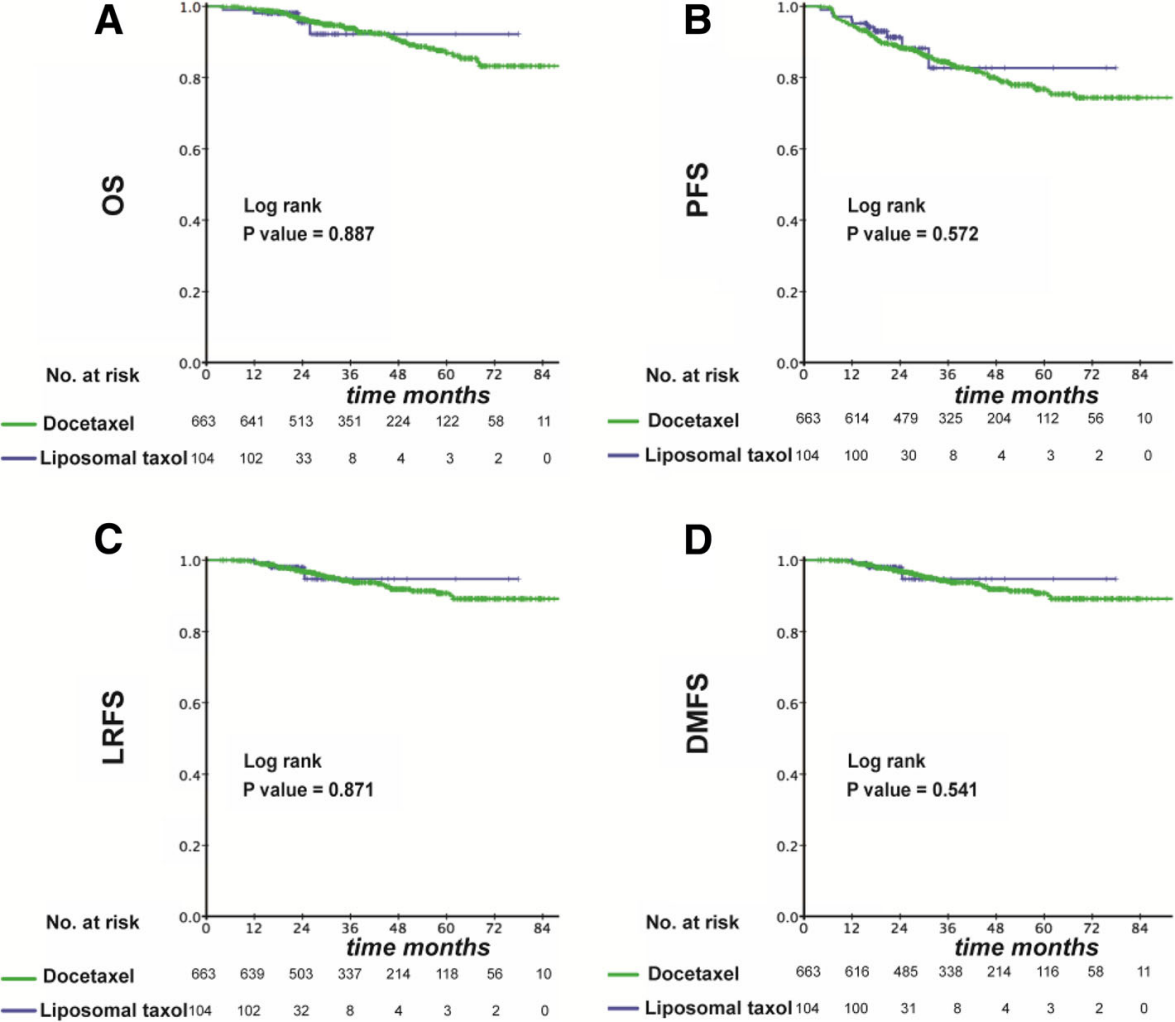
Kaplan–Meier curves in the initial cohort with a liposomal paclitaxel-based TPF regimen or docetaxel-based TPF regimen for OS (**a**), PFS (**b**), LRFS (**c**) and DMFS (**d**)

Among the original cohort of 767 patients, the median duration of follow-up was 21.5 months in the liposomal-paclitaxel group and 36.9 months in the docetaxel group, respectively. Overall, there was no significant difference in OS, PFS, LRFS and DMFS at 3 years between the two groups (OS: 92.2% vs. 93.9%, *P* = 0.887; PFS: 82.7% vs. 83.9%, *P* = 0.572; LRFS: 94.7% vs. 94.0%, *P* = 0.871; DMFS: 84.7% vs. 89.1%, *P* = 0.541) (Fig. 2a–d).

In multivariate analysis, the following variables were considered in the Cox proportional hazards model: age, sex, T stage, N stage, overall stage, level of EBV DNA, and chemotherapy regimen. As shown in Table 2, application of liposomal paclitaxel was not associated with a higher risk of death (hazard ratio (HR), 1.017; 95% confidence interval (CI), 0.361–2.868; *P* = 0.974), tumor progression (0.829; 0.429–1.603; 0.578), locoregional relapse (0.994; 0.301–3.288; 0.992) or distant metastasis (0.777; 0.353–1.708; 0.529) than application of docetaxel.

**Table 2:** Multivariable analyses of prognostic factors

	Observational dataset (*n* = 767)		PSM dataset(*n* = 390)	
Characteristic	Hazard ratio (95% CI)	*P*	Hazard ratio (95% CI)	*P*
Overall survival				
Age (≥45 vs. < 45)	1.342(0.797–2.258)	0.268	1.993 (0.894–4.440)	0.092
Sex (male vs. female)	1.111 (0.597–2.068)	0.739	0.873(0.365–2.087)	0.760
T stage (T3–4 vs T1–2)	1.336(0.561–3.181)	0.513	1.247(0.403–3.857)	0.701
N stage (N2–3 vs N0–1)	1.449(0.767–2.736)	0.253	0.959(0.364–2.525)	0.932
Overall stage((IVa–b vs. III)	1.093(0.640–1.869)	0.744	1.128(0.495–2.571)	0.774
EBV DNA(≥1500 vs. < 1500)	1.532(0.783–2.996)	0.213	1.393(0.638–3.043)	0.406
Regimen(Liposomal PTX vs. docetaxel)	1.017(0.361–2.868)	0.974	0.913(0.302–2.762)	0.872
Progression-free survival				
Age (≥45 vs. < 45)	0.871(0.609–1.246)	0.450	0.990 (0.597–1.642)	0.970
Sex (male vs. female)	2.150(0.977–1.509)	0.004	1.959 (0.991–3.875)	0.053
T stage (T3–4 vs T1–2)	0.960(0.559–1.649)	0.883	0.856(0.441–1.659)	0.644
N stage (N2–3 vs N0–1)	1.338(0.858–2.085)	0.199	1.096(0.552–2.176)	0.793
Overall stage((IVa–b vs. III)	1.016(0.705–1466)	0.930	0.858(0.515–1.430)	0.557
EBV DNA(≥1500 vs. < 1500)	1.341(0.879–2.044)	0.173	1.453(0.886–2.382)	0.138
Regimen(Liposomal PTX vs. docetaxel)	0.829 (0.429–1.603)	0.578	0.746(0.373–1.491)	0.407
Loco-regional relapse-free survival				
Age (≥45 vs. < 45)	0.895(0.489–1.638)	0.718	0.745(0.282–1.965)	0.552
Sex (male vs. female)	2.746(1.076–7.012)	0.035	1.768(0.509–6.142)	0.370
T stage (T3–4 vs T1–2)	1.847(0.557–6.127)	0.316	3.620(0.464–28.263)	0.220
N stage (N2–3 vs N0–1)	0.860(0.443–1.669)	0.655	1.063(0.330–3.427)	0.918
Overall stage((IVa–b vs. III)	0.851(0.462–1.567)	0.604	0.798(0.310–2.055)	0.640
EBV DNA(≥1500 vs. < 1500)	1.464 (0.710–3.020)	0.302	1.010(0.400–2.550)	0.983
Regimen(Liposomal PTX vs. docetaxel)	0.994(0.301–3.288)	0.992	1.027(0.285–3.702)	0.967
Distant metastasis-free survival				
Age (≥45 vs. < 45)	0.796(0.508–1.245)	0.317	0.917(0.499–1.685)	0.780
Sex (male vs. female)	1.894 (1.022–3.509)	0.042	1.665(0.768–3.613)	0.197
T stage (T3–4 vs T1–2)	0.816(0.434–1.536)	0.529	0.700(0.332–1.477)	0.349
N stage (N2–3 vs N0–1)	1.669(0.919–3.029)	0.092	1.312(0.533–3.228)	0.555
Overall stage((IVa–b vs. III)	1.061(0.672–1.674)	0.801	0.872(0.472–1.610)	0.661
EBV DNA(≥1500 vs. < 1500)	1.485(0.868–2.540)	0.149	1.879(1.032–3.423)	0.039
Regimen(Liposomal PTX vs. docetaxel)	0.777 (0.353–1.708)	0.529	0.694(0.304–1.587)	0.387

*CI* confidence interval, *EBV* Epstein–Barr virus

A Cox proportional hazards regression model was used to detect variables one by one without adjustment. All variables were transformed into categorical variables. HRs were calculated for age in years (≥45 vs. < 45); sex (male vs. female); T stage (3–4 vs. 1–2); N stage (2–3 vs. 0–1); overall stage (IVa–b vs. III); EBV DNA (≥1500 copies/mL vs. < 1500 copies/mL); type of treatment (liposomal paclitaxel vs. docetaxel)

**Fig. 3:**
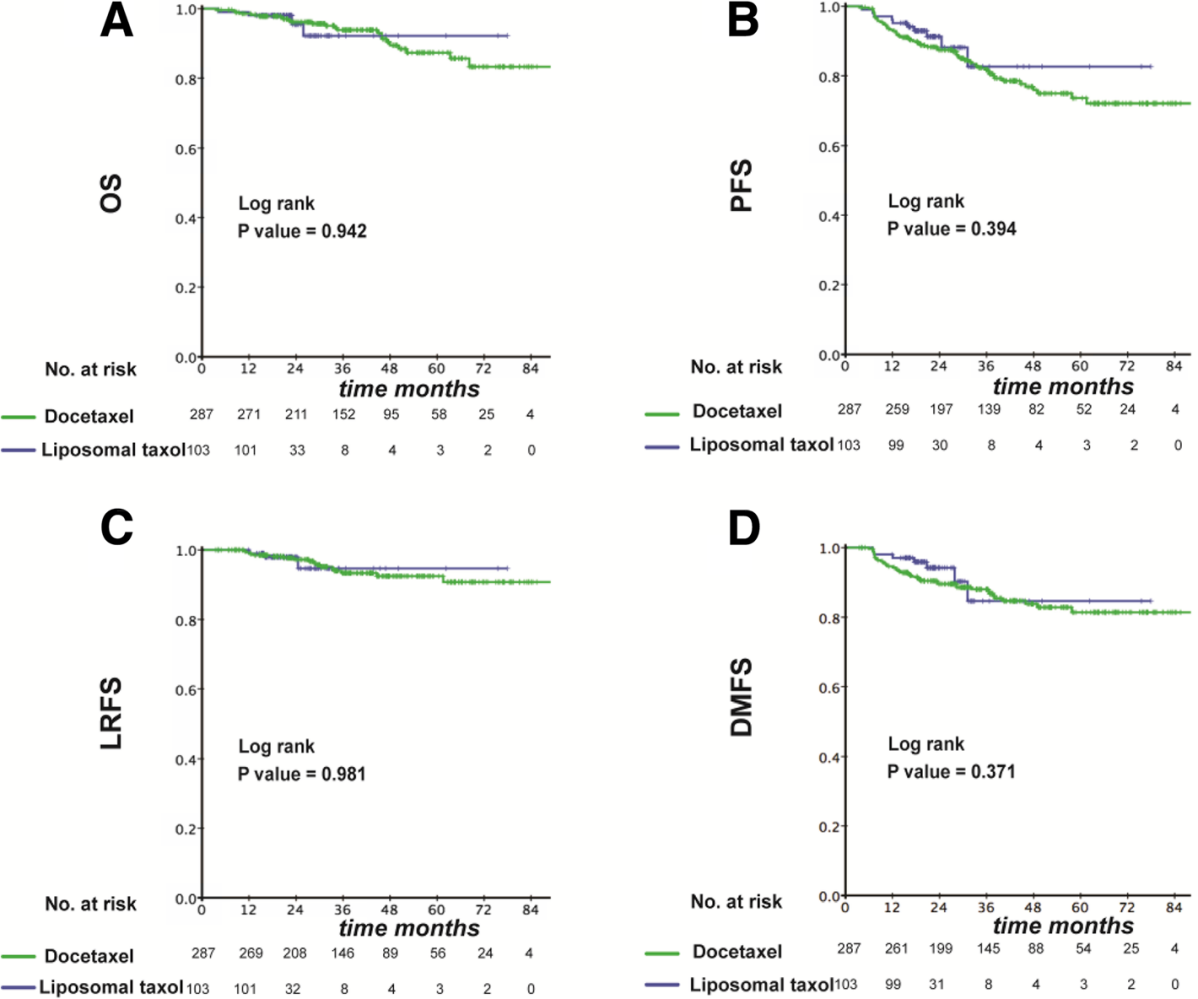
Kaplan–Meier curves in the PSM cohort with a liposomal paclitaxel-based TPF regimen or docetaxel-based TPF regimen for OS (**a**), PFS (**b**), LRFS (**c**) and DMFS (**d**)

In the propensity score-matched cohort of 390 patients, the median duration of follow-up was 21.6 months in the liposomal-paclitaxel group and 36.9 months in the docetaxel group, respectively. Similar to the result in original cohort, application of liposomal paclitaxel resulted in similar survival to that observed with docetaxel at 3 years (OS: 92.2% vs. 93.9%, *P* = 0.942; PFS: 82.6% vs. 81.7%, *P* = 0.394; LRFS: 94.7% vs. 93.3%, *P* = 0.981; DMFS: 84.6% vs. 87.4%, *P* = 0.371) (Fig. 3a–d). In multivariate analysis, all clinical endpoints were also highly independent from the selection of chemotherapy regimen (OS: HR, 0.913; 95% CI, 0.302–2.762; *P* = 0.872; PFS: 0.746; 0.373–1.491; 0.407; LRFS: 1.027; 0.285–3.702; 0.967; DMFS: 0.694; 0.304–1.587; 0.387) (Table 2).

### Acute toxicity

We evaluated acute toxicity during IC in the propensity score-matched cohort. No hypersensitivity reactions were observed in the liposomal-paclitaxel group. A liposomal paclitaxel-containing chemotherapy regimen was associated with a lower prevalence of grade-3–4 leukopenia significantly (grade 0–2: 96.1% vs. 70%; grade 3–4: 3.9% vs. 30.0%; *P* < 0.001) and neutropenia (grade 0–2: 78.6% vs. 53.0%; grade 3–4: 21.4% vs. 47.0%; P < 0.001) compared with the docetaxel-containing regimen. Intergroup differences in other acute toxicities such as anemia, as well as increases in levels of alanine transaminase, aspartate transaminase, and blood–urea–nitrogen were not significant (*P* > 0.05 for all) (Table 3).

**Table 3:** Grade-3–4 acute toxicities between the two groups of patients

Adverse event (toxicity grade)	Liposomal PTX (*n* = 103)		Docetaxel (*n* = 287)		*P*
	0–2 (%)	3–4 (%)	0–2 (%)	3–4 (%)	
Leukopenia	99(96.1)	4(3.9)	201(70.0)	86(30.0)	< 0.001^c^
Neutropenia	81(78.6)	22(21.4)	152(53.0)	135(47.0)	< 0.001^a^
Anemia	103(100)	0(0.0)	282(98.3)	5(1.7)	0.331^b^
Thrombocytopenia	103(100)	0(0.0)	283(98.6)	4(1.4)	0.577^b^
ALT increase	102(99.0)	1(1.0)	283(98.6)	4(1.4)	1.000^c^
AST increase	103(100)	0(0.0)	283(98.6)	0(0.0)	–
Creatinine increase	103(100)	0(0.0)	283(98.6)	0(0.0)	–
BUN increase	103(100)	0(0.0)	283(98.6)	0(0.0)	–
Nausea	101(98.1)	2(1.9)	279(97.2)	8(2.8)	1.000 ^b^
Vomiting	97(94.2)	6(5.8)	263(91.6)	24(8.4)	0.520 ^a^
Mucositis	103(100)	0(0.0)	286(99.7)	1(0.3)	1.000 ^b^
Neuropathy	103(100)	0(0.0)	287(100)	0(0.0)	–

*PTX* paclitaxel, *ALT* alanine aminotransferase, *AST* aspartate aminotransferase, *BUN* blood urea nitrogen

^a^*P* value was calculated using the chi-square test. ^b^*P* value was calculated using Fisher’s exact test. ^c^*P* value was calculated by correction for continuity chi-square test

## Discussion

Distant metastasis is a critical issue in advanced NPC [5, 6]. There is increasing evidence that IC can facilitate eradication of micro-metastatic lesions and reduce the risk of distant metastasis [7–9], so IC is being used widely in clinical settings. Based on studies that have established its superiority over the standard PF regimen for lowering the prevalence of distant metastasis and improving OS prevalence, TPF has emerged as the effective IC regimen [10–12].

In comparison with the standard PF regimen, the TPF regimen includes taxanes, effective antitumor drugs used for the treatment of different types of malignancies for decades. Taxanes bind preferentially to microtubules, leading to stabilization, and are involved in the formation of mitotic spindles during the M phase of the cell cycle [24–26]. Taxanes also induce apoptosis and have anti-angiogenic properties [27]. Paclitaxel, a taxane plant product, is one of the most commonly used broad-spectrum anti-cancer agents and has been approved for the treatment various cancers. However, the poor aqueous solubility and serious side effects associated with commercial preparations of paclitaxel has triggered the development of alternative paclitaxel formulations [28].

Docetaxel is a semi-synthetic taxane-based agent [14] which has supplanted conventional paclitaxel as the most commonly used paclitaxel-type drug used clinically. Compared with conventional paclitaxel, docetaxel has linear pharmacokinetics, a longer plasma half-life, and longer intracellular retention [27]. The TAX 323 study was the first to demonstrate the benefits of adding docetaxel to cisplatin and 5-fuorouracil as an IC for locoregionally advanced head-and-neck cancer [29]. Subsequently, the TAX 324 study [30, 31] and GORTEC laryngeal study [32] also showed that TPF was significantly better than PF at improving survival, local control, and organ preservation, and was associated with manageable toxicity. In addition, a randomized phase-III study conducted by Sun et al. [33] showed that TPF could increase 3-year failure-free survival for NPC patients significantly.

However, the main adverse effect of docetaxel is myelosuppression. Peng and colleagues showed that the prevalence of leukopenia and neutropenia using TPF was 28.1 and 45.0%, respectively [12]. A randomized phase-III study suggested that the most common grade-3 or − 4 adverse events during treatment in the TPF group were neutropenia (42%), leukopenia (41%) and stomatitis (41%) [33]. In our study, the prevalence of grade-3–4 leukopenia was 30.0% and that of neutropenia was 47.0% in the docetaxel-containing TPF regimen, a value that is consistent with that documented in previous studies.

Liposomal paclitaxel is a new paclitaxel drug encapsulated with liposomes. As new drug carriers, liposomes can improve the solubility of paclitaxel and prolong its action in vivo. Thus, liposomal formulations offer unsurpassed advantages, including the ability to carry a hydrophobic payload, ease of synthesis, favorable manufacturing control, and excellent biocompatibility [34–36]. As a result, liposomal paclitaxel can reduce toxicities, improve bioavailability [16, 17] and has comparable antitumor efficacy with conventional paclitaxel [18]. Huang and co-workers showed that liposomal paclitaxel exhibited a higher therapeutic index than clinical paclitaxel formulations [36].

Furthermore, hypersensitivity reactions are important adverse events in paclitaxel-based treatment, and can lead to death. Studies have shown that 8–14% of patients develop hypersensitivity reactions during paclitaxel-based chemotherapy [37, 38]. In our study, a hypersensitivity reaction was not observed, a result that is consistent with that in other studies [39–41]. The results shown above suggest that a liposomal paclitaxel-based TPF regimen is safe treatment for locally advanced NPC, especially for hypersensitive patients. In addition, the overall toxicity of liposomal paclitaxel was lower than that of free paclitaxel [42].

Su and colleagues showed that liposomal paclitaxel has the same anti-tumor efficacy as that of docetaxel and had greater safety for the treatment of breast cancer [43]. Lu and co-workers found that liposomal paclitaxel combined with capecitabine was, in general, well tolerated, and worth further study compared with docetaxel, cisplatin, and 5-fluorouracil in the treatment of gastric cancer [43, 44]. However, few studies have compared the efficacy and toxicity of liposomal paclitaxel with that of docetaxel.

This is the first study to show that application of liposomal paclitaxel results in comparable survival to that of docetaxel in NPC patients. Furthermore, consistent with other studies, patients in the liposomal-paclitaxel group were associated with a lower prevalence of grade-3–4 leukopenia and neutropenia in our study. Treatment goals for NPC are improvement of survival and reduction of treatment toxicity. Hence, selection of the treatment regimen should be based on drug efficacy and patient preference. Our study provides clinical evidence to support the use of a liposomal paclitaxel-based TPF regimen as an efficacious regimen for NPC.

Our study had three main limitations. First, as a retrospective study, there was an inevitable bias caused by selection. Second, data for hypersensitivity reactions due to docetaxel were not collected. Third, the OS value was limited because of the relatively short duration of follow-up; longer follow-up is needed to assess OS fully. Forth, liposomal paclitaxel patients were treated more recently and we did not include year of treatment in the PSM. In addition, 99% of the patients in our study were WHO III NPC and whether the results can be applied to western patients with WHO I/II NPC remains to be determined in future studies. Prospective studies are required to confirm our results.

## Conclusions

The present study suggests that application of liposomal paclitaxel results in comparable survival with that of conventional docetaxel with a lower prevalence of grade 3–4 leukopenia and neutropenia. A liposomal paclitaxel-based TPF regimen could be an alternative treatment strategy to a docetaxel-based TPF regimen in patients with locally advanced NPC.

## Abbreviations

CCRT: concurrent chemoradiotherapy
CIs: confidence intervals
DMFS: distant metastasis-free survival
EBV: Epstein–Barr virus
HR: hazard ratio
IC: induction chemotherapy
IMRT: intensity-modulated radiotherapy
LRFS: locoregional relapse-free survival
NPC: nasopharyngeal carcinoma
OS: overall survival
PF: cisplatin and 5-fluorouracil
PFS: progression-free survival
PSM: propensity score matching
RT: radiotherapy
TP: taxanes and cisplatin
TPF: taxanes, cisplatin and 5-fluorouracil
